# Focused ultrasound-mediated bbb disruption is associated with an increase in activation of AKT: experimental study in rats

**DOI:** 10.1186/1471-2377-10-114

**Published:** 2010-11-15

**Authors:** Shahrzad Jalali, Yuexi Huang, Daniel J Dumont, Kullervo Hynynen

**Affiliations:** 1 Molecular and Cellular Biology Research, Sunnybrook Research Institute, Sunnybrook Health Sciences Centre, Toronto, ON, Canada; 2 Department of Medical Biophysics, University of Toronto and Discipline of Imaging Research, Sunnybrook Health Sciences Centre, Toronto, ON, Canada

## Abstract

**Background:**

The Blood Brain Barrier (BBB) maintains the homeostasis of central nervous system by preventing the free passage of macromolecules from the systemic circulation into the brain. This normal physiological function of the BBB presents a challenge for delivery of therapeutic compounds into the brain. Recent studies have shown that the application of focused ultrasound together with ultrasound contrast agent (microbubbles) temporarily increases the permeability of the BBB. This effect is associated with breakdown of tight junctions, the structures that regulate the paracellular permeability of the endothelial cell layer. The influence of this ultrasound effect on the activation of intracellular signaling proteins is currently not well understood. Therefore, the aim of this study was to investigate the activation of cell survival signaling molecules in response to ultrasound-mediated BBB opening;

**Methods:**

The BBB was disrupted in two four-spot lines (1-1.5 mm spacing) along the right hemisphere of rat brain with ultrasound beams (0.3 MPa, 120 s, 10 ms bursts, repetition frequency = 1 Hz) in the presence *Definity *microbubbles. Contrast-enhanced MRI images were acquired to assess the extent of BBB opening upon which the animals were sacrificed and the brains removed and processed for biochemical and immunohistochemical analyses;

**Results:**

Immunoblotting of sonicated brain lysates resolved by SDS-PAGE demonstrated an increase in phosphorylation of Akt and its downstream signaling molecule, GSK3β, while the phosphorylation of MAPK remained unchanged. The elevated levels of pAkt and pGSK3β are still evident after 24 hours post-sonication, a time point where the integrity of the BBB is known to be re-established. Furthermore, immunofluoresence staining localized this increase in pAkt and pGSK3β levels to neuronal cells flanking the region of the disrupted BBB;

**Conclusions:**

Our data demonstrates that ultrasound-mediated BBB disruption causes an activation of the Akt signaling pathway in neuronal cells surrounding the disrupted BBB.

## Background

The homeostasis of the neuroparanchymal milieu is controlled by the presence of specialized tight junction structures existing between endothelial cells of brain microvessels. These cells are also in close contact with other cell types of the neural system, including astroctytes, pericytes, microglia and neurons. The complex signaling and communication which exists amongst these cell types gives rise to a barrier feature characterized as the Blood Brain Barrier (BBB). While the BBB restricts the paracellular translocation of large hydrophilic macromolecules and toxic compounds into the brain, there is a diffusion of lipid-soluble small molecules as well as facilitated passage of essential nutrients via specific membrane located transport systems[1-5]. However, the normal functions of the BBB are disturbed in neuroinflammatory conditions such as multiple sclerosis, Alzheimer disease (AD), HIV-1 encephalitis, traumatic brain injury, bacterial meningitis, brain tumors and ischemia/hypoxia [3,6,7].

While the regulation of the BBB function in normal and physiological conditions is very crucial, it hampers the effective delivery of therapeutic agents into the central nervous system. As a result, several strategies have been developed to overcome this impediment. For instance, intracarotid infusion of hypertonic solutions, like mannitol, has been shown to increase the permeability of the brain microvessels and promote access of therapeutic agents to the brain [8,9]. The transport of drugs from nasal mucosa to brain is another method to circumvent the BBB. It has been shown that the nasal cavity provides a special anatomical feature so that drugs can pass the olfactory epithelium and enter the central nervous system [10]. Although these modes of delivery into the brain are effective, they do not result in a focal disruption of the BBB for the treatment of localized diseases such as brain cancer.

Recently, it has been shown that the permeability of the BBB can be locally and temporarily disrupted by acoustic energy [11-13]. According to these studies, the application of focused ultrasound in combination with ultrasound contrast agents, such as Optison, can temporarily increase BBB permeability. As a consequence of this effect, the chemotherapeutic agents such as doxorubicin and Herceptin, can pass the barrier of endothelial cells and enter into the brain parenchyma at specific areas [11,12]. The significance of this method relies on opening of the BBB at specific locations of the brain while the rest of the areas remain unaffected.

The cellular and molecular mechanism underlying this effect is poorly understood. One report suggests that the oscillation of microbubbles in response to ultrasound bursts can induce cell membrane deformation and increase permeability of the endothelial cell monolayer [14]. In addition to trans-membrane mechanism, there is also an elevation in paracellular permeability of the endothelial cells and immunogold electron microscopy studies attribute this effect to disorganization as well as a diminished level of tight junction proteins occludin, claudin5 and ZO-1 [15]. Occludins and claudins are integral membrane tight junction proteins and their extracellular regions interact in a homotypic manner between two adjacent endothelial (or epithelial) cells. The intracellular domain of these proteins interact with cytoplasmic membrane-associated tight junction proteins called *zonula occludens *(ZO proteins), which act as a bridge between integral membrane proteins and the actin cytoskeleton [1,5]. As a result, any extracellular or intracellular signal imposed upon either the elements of tight junction or cytoskeletal proteins can have an effect on the integrity, tightness and assembly of the endothelial junctions and eventually the BBB [1,3]. To date, several cell signaling pathways, including MAPK and PI3kinase/Akt, are shown to play a role in regulation of brain endothelial cell permeability. It has been reported that reactive oxygen species (ROS) can alter the cytoskeletal as well as tight junctional organization via activation of PI3-kinase and Akt [16]. VEGF- and hypoxia-induced hyperpermeability of brain endothelial cells is also mediated by activation of PI3-Kinase [17]. Activation of MAPK signaling also contributes to the BBB opening in several pathological conditions like AD, focal ischemia and reperfusion, traumatic brain injury and subarachnoid hemorrhage (SAH) brain injury [18-20]. In vitro studies also show that H_2_O_2_-mediated increased paracellular permeability in hypoxia/reoxygenation is mediated by activation of p44/42 MAPK [21]. In addition to endothelial cells, PI3kinase/Akt and MAPK signaling play a role in regulating neuronal cells survival during pathological insults to the brain and disruption of the BBB. For example, it has been shown that phosphorylation of Akt after stroke results in neuronal survival [22]. The well-known vascular permeable factor or VEGF is known to increase the BBB permeability and enhances neuronal protection, the effects of both mediated by increased PI3-kinase/Akt activity [23]. Moreover, Neuroprotective effect of some hormones and peptides are mediated via increased phosphorylation and activation of p44/42 MAPK [24-27]. In contrast to p44/42 MAPK, increased phosphorylation and activation of p38 and c-Jun N-terminal kinase (JNK) members of MAPK promote the apoptosis of cerebella neurons [28-31].

To date there is no data available on the effect of ultrasound on cell signaling pathways in brain endothelial and neuronal cells. Therefore the aim of this study is to investigate the alteration in PI3kinase/Akt and MAPK signaling pathway in the context of focused ultrasound-mediated BBB opening.

In the following study we show that the application of focused ultrasound in combination with ultrasound contrast agent disrupts the BBB integrity, and that this disruption is associated with reduced interaction of the tight junction protein occludin with ZO-1. Furthermore, there is an increase in the activity of Akt signaling upon ultrasound exposure. The Akt signaling remains active even after 24 hours post-sonication, when the BBB integrity is re-established. Immunofluorescent microscopy demonstrates that the elevated pAkt as well as pGSK3β phosphorylation could be attributed to neuronal cells. In contrast to Akt signaling, ultrasound has no effect on the activity of MAPK pathway.

## Materials and Methods

### Animal Procedures

All animal experiments were performed on a total of 20-25 adult male Sprauge-Dawley rats weighing between 300-400 g and the procedures were approved by Animal Committee at our institution. Animals underwent anesthesia using a mixture of 50 mg/kg ketamine and 10 mg/kg xylazine. The hair layer on top of the skull was removed using depilatory lotion and a catheter was inserted into the tail vein of each rat. For macroscopic visualization of BBB opening, animals were injected with Evans Blue dye (500 μl of 2%) post-sonication.

### Ultrasound

The focused ultrasound system was placed inside a 1.5T MRI scanner (Signa, GE Healthcare, USA) that was used to image the brain and target the ultrasound beam. The ultrasound beam was generated by a piezoelectric transducer (10 cm diameter, 8 cm radius of curvature, 0.558 MHz resonant frequency, manufactured in house) positioned inside a degassed water tank using an MRI-compatible three-axis motorized system (Chopra et al., 2009). The transducer was driven by a function generator (Model 395, Wavetek, San Diego, CA, USA) and RF amplifier (model 240L, ENI Inc, Rochester, NY, USA). The electrical power was measured with a power meter (model 438A, Hewlett Packard, Palo Alto, CA, USA) connected to the forward and reserve signal of a dual directional coupler (C173, Werlatone, Brewster, NY, USA). The transducer's electrical impedance was matched to the output impedance of the amplifier (50Ω) with a custom-made passive matching circuit. The transducer was calibrated using a radiation force method for the acoustic power and calibrated hydrophone for the acoustic pressure amplitude. Four spots, 1.5 mm apart, along the right hemisphere were targeted with ultrasound (0.3 MPa, 120 s, 10 ms bursts, repetition frequency = 1 Hz). Two four-spot lines (1-1.5 mm spacing) were applied to cover most of the area of the right hemisphere. *Definity *microbubbles (20 μl/kg, Lantheus Medical Imaging, USA) was injected simultaneous to the start of the sonication.

### MRI

Anatomical images were acquired in multiple planes prior to and after sonication using a T2-weighted sequence (Fast Spin Echo (FSE) sequence: TE = 75 ms, TR = 2000 ms, ETL = 4, BW = 6.9 kHz, 256 × 256/128 × 128, slice = 1 mm, NEX = 2, FOV = 5 cm) to evaluate whether signs of tissue damage were present after the exposures. After all sonications, a bolus injection of MR contrast agent (0.1 mmol/kg, Omniscan, GE Healthcare), was administered, followed by a saline flush, in order to visualize BBB disruption in the brain. Contrast-enhanced images were acquired using a T1-weighted imaging sequence (FSE, TE = 14 ms, TR = 500 ms, ETL = 4, BW = 15 kHz, 256 × 256/128 × 128, slice = 1.5 mm, NEX = 3, FOV = 5 cm) to evaluate the presence and extent of BBB opening.

### Immunoblot analysis

Sonicated brain regions defined with trypan blue leakage, and their counterparts from opposite brain hemispheres were isolated and homogenized in ice-cold RIPA lysis buffer (containing 50 mM Tris-HCl pH 7.4, 150 mM NaCl, 1 mM EDTA, 1 mM EGTA, 1 mM NaF, 20 mM Na_4_P_2_O_7_, 2 mM Na_3_VO_4_, 10% glycerol, 0.1% SDS, 1% Triton-X-100, 0.5% Deoxycholate and protease inhibitor cocktail). The homogenates were centrifuged at 15,000 g for 30 min at 4°C and the protein concentration of the supernatants was determined using BCA™ protein assay kit. 30 μg of total protein was mixed with 3× SDS sample buffer (containing 240 mM Tris-Hcl pH 6.8, 6% SDS, 30% glycerol, 16% β-mercaptoethanol and 0.06% Bromphenol Blue), boiled at 100°C for 5 min and resolved either in 10% or 7.5% SDS-PAGE gel. Separated proteins were wet transferred to PVDF membrane, blocked with 5% milk powder and consecutively incubated with primary antibodies overnight at 4°C and HRP-conjugated secondary antibodies 1 hour at room temperature. Protein signals were detected using Supersignal^® ^West Pico Chemiluminescent Substrate (Thermo Scientific) and the intensity of the bands was quantified using GeneSnap from SynGene software. Primary antibodies were: rabbit polyclonal anti-ZO-1 (Invitrogen, 5:1000), rabbit polyclonal anti-occludin (Invitrogen, 5:1000), mouse monoclonal anti-actin (Sigma), rabbit monoclonal anti-phospho-Akt (ser473), rabbit monoclonal anti-phospho-Akt (Thr308), rabbit monoclonal anti-Akt, rabbit monoclonal anti-phospho-GSK3β (ser9), rabbit monoclonal anti-phospho-p44/42 MAPK (Thr202/Tyr204), rabbit monoclonal anti-phospho-p44/42 MAPK, rabbit monoclonal anti-phospho-SAPK/JNK (Thr183/Tyr185), rabbit monoclonal anti-SAPK/JNK (all from Cell Signaling, 1:1000).

### Immunoprecipitation

Brain tissues were homogenized in ice-cold Immunoprecipitation (IP) buffer (containing 50 mM Tris-HCl pH 7.5, 150 mM NaCl, 2 mM EDTA, 10% glycerol, 10 mM sodium pyrophosphate, 10 mM β-glycerophosphate, 10 mM NaF, 1 mM sodium vanadate, 1% Nonidet p-40 and protease inhibitor cocktail). The homogenates were centrifuged at 15,000 g for 30 min in cold room and the supernatants were collected and subjected to protein assay as described in the previous section. For immunoprecipitation, supernatants containing 1 mg total protein in 500 μl IP buffer were first precleared with protein A Sepharose beads (GE Health care) for 1 hour and then incubated with 5 μg rabbit anti-occludin antibody (Invitrogen) overnight at 4°C, while constantly shaking on a rotator. The samples were then incubated with protein A Sepharose beads for 4 hrs at 4°C, and the formed immunocomplexes (protein+primary antibody+beads) were precipitated using centrifugation at 250 g for 1 min. The precipitates were washed three times with IP buffer, mixed with 3× SDS sample buffer, boiled for 5 min and then analyzed with immuno blotting as explained previously.

### Immunofluorescence Staining

For immunofluorescence staining of the brain sections, rats were transcardially perfused with ice-cold PBS and 4% paraformaldehyde. Perfusion was performed using a pump at a rate of 2.5 ml/min and the procedures included: 1) Clamping of abdominal aorta 2) Insertion of the perfusion syringe into the aorta through left ventricle 3) Drainage of the solutions through right atrium. After perfusion, the rats were decapitated and the brains were removed from the skulls. The brains were subsequently post-fixed in 4% paraformaldehyde for 24 hours followed by cryoprotection in 30% sucrose at 4°C overnight. The brain tissues were then paraffin-embedded using standard histological procedures and 7 μM-thick sections were prepared and adhered to VWR^® ^micro slides (Superfrost^® ^Plus). Immunofluorescence staining with specific antibodies was performed according to manufacturer's instruction. Briefly, sections were deparaffinized with xylene and then rehydrated with 100% and 95% ethanol respectively. After washing with dH_2_O, the antigens were unmasked by incubation of the sections at 10 mM sodium citrate buffer (pH 6) and heating them just below the boiling temperature (95-99°C) for 20 min. The slides were then cooled down at room temperature for 30 min, rinsed in dH_2_O and PBS respectively and then followed with immunostaining. The sections were blocked with blocking buffer (PBS/0.3% Triton-X-100/5% goat serum) for an hour at room temperature and then incubated with primary antibodies diluted with antibody dilution buffer (PBS/0.3% Triton-X-100/1% BSA) overnight at 4°C. After washing sections with PBS, they were incubated with Cy3-conjugated secondary antibody (Jackson ImmunoResearch Laboratories, 1:100) for 1 hour at dark and room temperature. Sections were then washed and covered with vectashield mounting medium containing DAPI and cover slips. The primary antibodies were: rabbit anti-phospho-Akt (Ser 473) (Cell signaling, 1:50), rabbit anti-phospho-GSK3β (ser9) (cell signaling, 1:100), Alexa Fluor^® ^488-conjugated Glial Fibrillary Acidic Protein (GFAP) mouse monoclonal antibody (Cell signaling, 1:100), Alexa Fluor^® ^488-conjugated Neuronal Nuclei (NeuN) mouse monoclonal antibody (Millipore, 1:100), rabbit anti-ZO-1 (Invitrogen, 1:100) and rabbit anti-Von Willebrand Factor (vWF) (Abcam, 1:500). The final fluorescent images were captured using ZEISS Axiovision 200 fluorescence microscope.

### Statistical Analysis

Statistical comparison between two groups was analyzed using ANOVA with Student t-test. The results were expressed as mean ± SE. *P *value of < 0.05 was statistically considered significant.

## Results

### Extravasation of IgG and enhancement of MRI contrast agent

**Figure 1:**
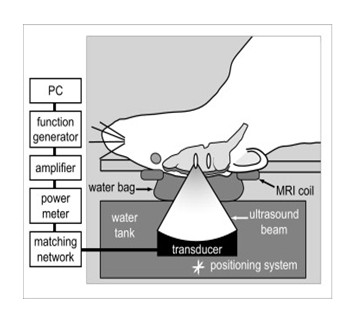
**Experiment set-up**. Anesthetized rats are placed on their backs with their heads on a water bag, which is placed above the ultrasound transducer. A MRI coil surrounds the head of the rat allowing for real-time imaging of the break-down of the blood brain barrier.

**Figure 2:**
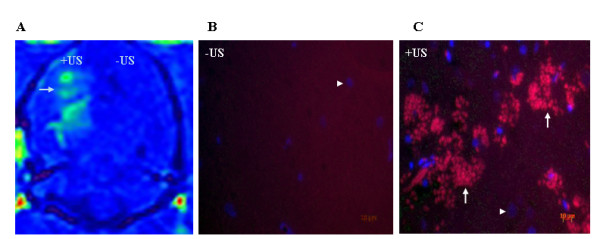
**Increased leakage of MRI contrast agent and IgG into the brain parenchyma as a result of ultrasound-mediated Blood Brain Barrier (BBB) disruption**. **A**, MRI image shows enhancement of the MRI contrast agent in the right hemisphere of the brain (+US) shortly upon exposure to ultrasound in the presence of microbubbles (green color, arrow). **B**, Immunofluorescence staining using cy3-conjugated anti-rat IgG shows IgG extravasation into the brain parenchyma as a result of BBB opening (+US, arrow). DAPI represents nuclei of the cells (arrow heads). '-US': refers to left hemisphere without ultrasound exposure. '+US': refers to right hemisphere with ultrasound exposure and disrupted BBB.

Application of focused ultrasound (Figure 1) in the presence of ultrasound contrast agent (microbubbles) increases the permeability of the BBB, the effect of which is shown by enhancement of MRI contrast agent (Figure 2A, arrow). To further determine the areas of leaky vessels, we examined the presence of extravasated blood components, such as IgG, in the regions of brain treated with ultrasound (+US). Immunofluorescence staining of non-sonicated brain tissue sections with cy3-conjgated anti-rat IgG antibody show no staining (Figure 2B, -US). While sections taken from sonicated regions show extensive staining for extravasated IgG (Figure 2C, +US, arrow).

### Diminished interaction of Zo-1 with occludin in response to ultrasound

**Figure 3:**
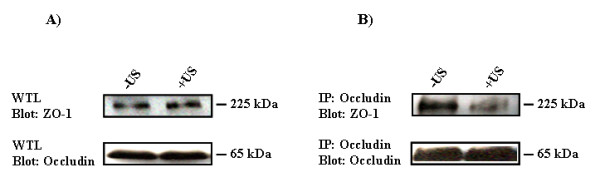
**Diminished interaction of ZO-1 and occludin as a result of ultrasound treatment**. Rat brain homogenates were prepared from both non-sonicated (-US) and sonicated (+US) regions. **A**, Western Blot analysis on whole brain tissue lysates (WTL) shows that ZO-1 and occludin protein levels are not changed in response to ultrasound treatment. **B**, Co-immunoprecipitation of occludin and ZO-1. The amount of Occludin co-precipitating with ZO-1 in the presence of US-treatment is reduced when compared to non-sonicated brains (-US).

The extracellular domains of occludin form homotypic interactions with adjacent cells. The intracellular portion of occludin interacts with the intracellular scaffolding proteins ZO-1 and ZO-2, providing a link to the cytoskeletal protein actin, which establishes the tight junction structure. A recent study by Sheikov et al [15] showed that application of focused ultrasound in the presence of ultrasound contrast agent resulted in increased paracellular permeability in brain vascular endothelium. Furthermore, immunoelectron microscopy showed that this effect is associated with delocalization as well as diminished immunogold signals of tight junctional proteins, including occludin, claudin 5 and ZO-1 [15]. Western blot analysis with brain tissue homogenates show that the protein levels of the tight junction proteins ZO-1 and occludin in the ultrasound treated region is not changed compared to non-treated regions (Figure 3A). However, using immunoprecipitation analysis we show that the interaction of occludin and ZO-1 is reduced in the brain regions treated with ultrasound (Figure 3B).

### Focused ultrasound treatment in the presence of ultrasound contrast agent, increases PI3 kinase/Akt signaling activity, while the activity of MAPK signaling pathways remains unaffected

**Figure 4:**
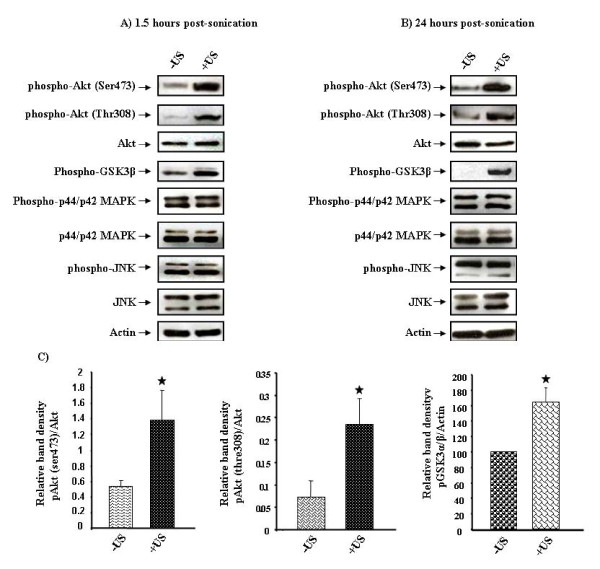
**Ultrasound in the presence of microbubbles increases the activity of the Akt signalling pathway, while the activity of MAPK signalling remains unchanged**. Brains were removed 1.5 hrs **(A) **or 24 hours **(B) **post-sonication and snap-frozen using liquid nitrogen. Brain tissue regions with trypan blue leakage in the sonicated hemisphere (+US) and the equivalent area from opposite hemisphere (-US) were then homogenized with RIPA lysis buffer. Equal amounts of extracted proteins were analyzed by western blotting for the indicated proteins. **C**, Graphical representation of three independent experiments illustrating the marked increase in pAkt (Ser473), pAkt (Thre308) and pGSK3β (Ser9) 1.5 hrs after sonication treatment. The final data were analyzed with student's t-test. Error bars indicate mean ± SE and the star (*) represents p < 0.05 for -US versus +US.

PI3kinase/Akt signaling has been shown to be responsible for both vascular permeability and neuronal survival [23]. In addition, both *in vivo *and *in vitro *studies have suggested a role for the MAPK kinase signaling pathway in controlling the permeability of the BBB [18-21]. We investigated the potential modulation of Akt and MAPK signaling activity. We show that phosphorylation of Akt at both serine (S473) and threonine (T308) residues is increased in the sonicated brain, while the total Akt protein level remains unchanged (Figure 4A). Moreover, the downstream signaling molecule of Akt, GSK3β, is highly phosphorylated, further indicating the activation of Akt signaling pathway in response to ultrasound treatment (Figure 4A). Quantification of western blot band densities indicates that this increase is significantly (P < 0.05) higher in the sonicated rat brain areas as compared to non-treated contralateral regions (Figure 4C). The increased phosphorylation of Akt and GSK3β is also observed after 24-hours post-sonication (Figure 4B). In contrast, there is no alteration at both phosphorylated and non-phosphorylated levels of MAPK signaling molecules (Figure 4A and 4B).

### Increased p-Akt and pGSK3β localizes to neuronal cells flanking the disrupted BBB

**Figure 5:**
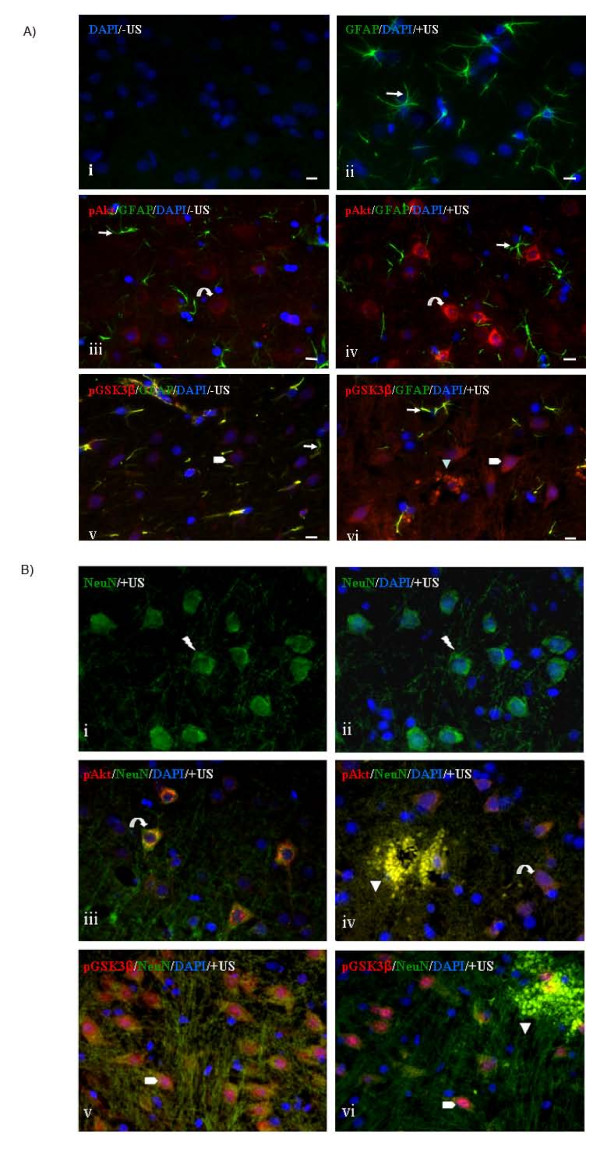
**Increased phosphorylation of Akt and GSK3β in neuronal cells of sonicated rat brain regions**. Paraffin sections of rat brains were immunofluorescently stained with primary antibodies against astrocyte marker, Glial Fibrillary Protein (GFAP, green), Neuronal marker (NeuN, green), phospho-Akt (pAkt, red) and phospho-GSK3 β (pGSK3β, red). In all sections, DAPI (blue) represents nuclear staining of the cells. **A**, Panel [**i**] shows a control section without application of any primary antibodies. Panel [**ii**] represents a direct staining of the astrocytes with Alexa-fluor488-conjugated GFAP (green, right arrow). Panels [**iii**] and [**iv**] represent immunofluorescence staining with pAkt (red, curved arrow) and GFAP (green, right arrow) in non-sonicated (-US) as well as sonicated (+US) hemispheres respectively. Panels [**v**] and [**vi**] illustrate co-staining of pGSK3β (red, pentagon) and GFAP (green, right arrow) in '-US' and '+US' brain regions respectively. **B**, Panels [**ii**] and [**iii**] represent neuronal cells morphology as directly stained with Alexa-Fluor 488-conjugated NeuN (green, lightning bolt). Co-staining of pAkt (red, curved arrow) with NeuN (green) and also pGSK3β (red, pentagon) with NeuN (green) are shown in panels [**iii, iv**] and [**v, vi**] respectively. The regions of the brain sections with IgG extravasation (arrow head) and their surrounding neuronal cells with elevated levels of pAkt and pGSK3β are shown in panels [**iv, vi**].

Ultrasound-mediated disruption of the BBB is associated with increased phosphorylation of Akt and its downstream signaling molecule GSK3β as shown in Figure 4A and Figure 4B. The elevated pAkt and pGSK3β levels extend until 24 hours post-sonication, when the integrity of the BBB is known to be re-established [15]. To investigate the origin of the cell types with increased Akt activity, paraffin sections of brain tissues were immuono-labelled with either pAkt or pGSK3β together with either neuronal (NeuN) or astrocyte marker (GFAP). The morphology of the cells is illustrated with single staining of either GFAP (Figure 5A [ii]) and NeuN (Figure 5B [i, ii]). Cells with increased pAkt (Figure 5A [iv] and Figure 5B [iii, iv]) and pGSK3β (Figure 5A [vi] and Figure 5B [v,vi]) are shown in sonicated brain areas. Fluorescent co-staining shows that the increased pAkt and pGSK3β are localized to neuronal cells of sonicated brain areas (Figure 5B [iii, iv, v, vi]). Figure 5B [iv, vi] displays the neuronal cells and their nearby extravasated IgGs, where there is an increased BBB permeability.

## Discussion

The BBB restricts the free passage of the macromolecules from peripheral circulation to the central nervous system and consists of the endothelial cells adhered to one another via elaborate tight junction structures. The physiological properties of the BBB are highly regulated by a wide variety of the signals originating either from luminal (blood) or abluminal (astrocytes, pericytes, neurons) face of the brain microvessel endothelial cells [1,33-35]. While being inherently important, the normal barrier function of the BBB is challenging when dealing with brain delivery of therapeutic agents and requires strategies to temporarily increase the permeability of the brain endothelial cells. Recently, it has been shown that focused ultrasound in the presence of circulatory microbubbles (ultrasound contrast agent) is able to locally and temporarily disrupt the BBB, providing a potential and promising method for drug delivery [11,12,15]. In the present study, the immunofluorescence staining of rat IgG in the sonicated brain areas further confirms the increased permeability of endothelial cells and disruption of the BBB.

Focused ultrasound-mediated increase in paracellular permeability has been described as one of the entry routes of macromolecules into the brain and immunogold electron microscopy studies have shown that this increase is associated with disarrangement and reduced immunogold signals of tight junction proteins including occludin, claudin 5 and ZO-1 [15]. The diminished level of immunogold signals at the junction could be due to degradation or the intracellular redistribution of tight junction proteins. Here we show that the protein levels of occludin and ZO-1 are not altered, but instead their interaction is considerably reduced in the sonicated brain areas, suggesting that the decrease in immunogold signals may be the consequence of the redistribution of tight junction proteins. The interaction of occludin and ZO-1 is required for proper functioning of the tight junctions and is also subject to tight regulation by several mechanisms [1,35]. It has been shown that the post-translational modifications play an essential role in controlling the interaction of occludin and ZO-1. There are several reports indicating that the phosphorylation of occludin at tyrosine residues (Tyr-398 and Tyr-402) decreases the interaction of this protein with ZO-1, ZO-2 and ZO-3, thus enhancing the permeability of the blood vessels [36,37]. Increased phosphorylation of occludin at tyrosine residue is shown in a focal cerebral ischemia model of the BBB disruption, further supporting a role for tight junction protein phosphorylation in regulating the permeability of blood vessels [38]. Occludin can also become phosphorylated on serine and threonine (ser/thre) residues, but the effect of this phosphorylation event is distinct from that of tyrosine residue. In fact, elevated ser/thre phosphorylation of occludin promotes the assembly of the tight junctions, resulting in a reduction in permeable vessels [39]. Given that the phosphorylation of the proteins is an immediate event and the disruption of the BBB also occurs shortly after sonication, it is plausible that the ultrasound-mediated diminished interaction of occludin and ZO-1 is due to alteration in phosphorylation level of tight junction proteins.

The synthesis, assembly and function of tight junction proteins and their ultimate effect on BBB opening, could be influenced by several signaling molecules. For instance, Rho signaling, Myosine Light Chain Kinase (MLCK), PKC, PKA, PKG, phosphatases (PP1, PP2A and PP2B), MAPK and PI3kinase/Akt pathways are shown to be implicated in the regulation of tight junction function and vessel leakage [33]. In this study we investigated a role for PI3 kinase/Akt and MAPK signaling in response to focused ultrasound-mediated BBB disruption. The PI3kinase/Akt signaling pathway is shown to be activated by a number of cell-membrane located receptors (G-protein-coupled receptors and tyrosine kinases) and plays an important role in the survival of both endothelial and neuronal cells [40]. Both *in vivo *and *in vitro *studies suggest that the increased Akt activity is involved in hyperpermeability of the BBB [16,17,23]. Furthermore, ultrasound application is known to produce shear stress [41]. Given that shear stress also triggers the activation of Akt in vascular endothelial cells [42], we might assume that ultrasound treatment triggers activation of Akt, leading to increased permeability of the endothelial cells. In addition, active Akt can phosphorylate endothelial nitric oxide synthase (eNOS), the activity of which increases NO production and the BBB permeability [43]. In the current study we show that both Akt and GSK3β phosphorylation are increased in the ultrasound-received brain regions, implying that increased Akt activity might contribute to the BBB opening. However, increased phosphorylation of Akt is also evident 24 hours after sonication, the time window that the tight junction proteins are re-assembled and the BBB function is restored.

Our immunofluorescence staining localizes increased pAkt and pGSK3β levels into neuronal cells of the BBB-disrupted brain areas, implying that ultrasound application results in the activation of survival pathways in neuronal cells. This observation is in accordance with a previous report indicating that in a cerebral ischemia model of the BBB disruption, the increased PI3kinase/Akt signaling promotes neuronal survival [22]. Akt is a downstream signaling molecule of tyrosine kinase receptors and is activated upon binding of these receptors to their cognate ligand molecules [40]. VEGF is one of the signaling molecules that binds to its receptors on cell membrane, VEGFR1 and VEGFR2, initiating signaling pathways through Akt activation. Transgenic animals expressing human VEGF_165 _under a neuron-specific promoter, as well as intracerebroventricular administration of VEGF both are shown to increase Akt activity and enhance neuronal survival after focal cerebral ischemia [23,44]. It is plausible that the ultrasound-mediated increase of neuronal Akt activity is the result of VEGF action. In addition to VEGF, there might be other factors which could induce the elevation of Akt in neuronal cells. For instance, the extravasation of blood components into the brain can initiate several signaling pathways in the neuronal and glial cells. As a result of the BBB disruption, fibrinogen is shown to enter the brain parenchyma, where it binds and induces the phosphorylation of epidermal growth factor receptor (EGFR) in an integrin- and src-dependent manner [45]. Given that tyrosine kinase EGFR is upstream signaling molecule of Akt, it is possible that extravasated fibrinogen turns on the activity of Akt in the sonicated brain regions.

## Conclusions

Our data provides evidence that focused ultrasound in the presence of circulating ultrasound contrast agents (microbubbles) disrupts the interaction of tight junction proteins (occludin and ZO-1), thus affecting the integrity of the BBB. Application of ultrasound results in activation of PI3kinase/Akt signaling pathway in neuronal cells.

## Competing interests

KH is an inventor in patents describing this technology. DJD, YH & JJ declare that they have no competing interests.

## Author contributions

SJ carried out the biochemical experiments and wrote the manuscript. YH performed the ultrasound treatments. DJD provided guidance in project design and proofed the manuscript. KH directed the design of the ultrasound aspects of the project, supervised the ultrasound treatments and proofed the manuscript.

## Pre-publication history

The pre-publication history for this paper can be accessed here:

http://www.biomedcentral.com/1471-2377/10/114/prepub
